# Comprehensive analysis of immune-related biomarkers and pathways in intracerebral hemorrhage using weighted gene co-expression network analysis and competing endogenous ribonucleic acid

**DOI:** 10.3389/fnmol.2022.955818

**Published:** 2022-09-26

**Authors:** Yuehan Hao, Xiaoxue Xu, Yuye Wang, Feng Jin, Ling Tang, Wenxu Zheng, Heyu Zhang, Zhiyi He

**Affiliations:** ^1^Department of Neurology, The First Affiliated Hospital of China Medical University, Shenyang, China; ^2^Department of Geriatric, Dalian Friendship Hospital, Dalian, China; ^3^Department of Neurology, The First Affiliated Hospital, Sun Yat-sen University, Guangzhou, China; ^4^Guangdong Provincial Key Laboratory of Diagnosis and Treatment of Major Neurological Diseases, National Key Clinical Department and Key Discipline of Neurology, Guangzhou, China

**Keywords:** ICH, immune-related, ceRNA, WGCNA, lncRNA

## Abstract

The immune response is an important part of secondary brain injury following intracerebral hemorrhage (ICH), and is related to neurological deficits and prognosis. The mechanisms underlying the immune response and inflammation are of great significance for brain injury and potential functional restoration; however, the immune-related biomarkers and competing endogenous ribonucleic acid (RNA) (ceRNA) networks in the peripheral blood of ICH patients have not yet been constructed. We collected the peripheral blood from ICH patients and controls to assess their ceRNA profiles using LCHuman ceRNA microarray, and to verify their expression with qRT-PCR. Two-hundred-eleven DElncRNAs and one-hundred-one DEmRNAs were detected in the ceRNA microarray of ICH patients. The results of functional enrichment analysis showed that the immune response was an important part of the pathological process of ICH. Twelve lncRNAs, ten miRNAs, and seven mRNAs were present in our constructed immune-related ceRNA network, combining weighted gene co-expression network analysis (WGCNA). Our study was the first to establish the network of the immune-related ceRNAs derived from WGCNA, and to identify leukemia inhibitory factor (LIF) and B cell lymphoma 2-like 13 (BCL2L13) as pivotal immune-related biomarkers in the peripheral blood of ICH patients, which are likely associated with PI3K-Akt, the MAPK signaling pathway, and oxidative phosphorylation. The MOXD2P-miR-211-3p -LIF and LINC00299-miR-198-BCL2L13 axes were indicated to participate in the immune regulatory mechanism of ICH. The goal of our study was to offer innovative insights into the underlying immune regulatory mechanism and to identify possible immune intervention targets for ICH.

## Introduction

Intracerebral hemorrhage (ICH) accounts for ∼10–20% of strokes and has a higher rate of mortality and disability than ischemic stroke (An et al., 2017). The 1-year survival rate of ICH is ∼40%, and the 10-year survival rate is ∼24% (Gross et al., 2019). Survivors have different degrees of neurological dysfunction and complications, such as paralysis, symptomatic epilepsy, and dementia (Cordonnier et al., 2018), leading to a huge economic impact and social burden. At present, the therapeutic methods of ICH mainly include hemostatic agents, reducing intracranial pressure, controlling blood pressure within the normal range, surgery, and symptomatic treatments. However, there is no specific targeted therapy for ICH that can improve prognosis.

The immune response is an important part of secondary brain injury following ICH, and can lead to neurological deficits and affect patient prognosis. Following ICH, microglial activation and death cells products trigger an inflammation response and destroy the blood brain barrier. Then, monocytes, macrophages, neutrophils, and T cells promote the inflammatory process around the hematoma (Mracsko and Veltkamp, 2014; Fu et al., 2015; Shtaya et al., 2021). The mechanism underlying the immune and inflammation responses are of great significance for brain injury and potential functional restoration. Immune intervention, which is currently being explored, may reduce damage to the brain caused by the immune response, and is expected to become an effective remedy for ICH.

Long non-coding RNAs (lncRNAs) of > 200 nucleotides share similar functions with mRNAs in biogenesis and transcript regulation (Deng et al., 2019; Zhu et al., 2020; Xie et al., 2021). Most lncRNAs do not translate into proteins; only some encode small peptides (Kopp and Mendell, 2018; Fan et al., 2020). In addition, lncRNAs can sponge microRNAs (miRNAs), also known as competing endogenous RNAs (ceRNAs), to recognize cytoplasmic miRNAs and isolate them from mRNAs, thereby regulating the expression of protein targeted by miRNAs. At present, studies on immune-related lncRNAs in ICH are limited to particular lncRNAs or animal models. For example, lncRNA Blnc1 was up-regulated in ICH mouse brains with hematoma edema, and induced apoptosis, permeability, and an inflammatory response (Xie et al., 2021). Another study reported that high expression of lncRNA Ptprj-as1 in an ICH rat model promoted the migration of BV2 cells, increased the proportion of M1 glial cells, stimulated the secretion of inflammatory cytokines, and participated in the inflammatory injury caused by ICH (Wen et al., 2018). However, a study on the immune-related lncRNA-miRNA-mRNA ceRNA network in ICH patients had not yet been reported.

In this study, the peripheral blood from ICH patients and control subjects was collected for LCHuman ceRNA microarray and verification using qRT-PCR. We detected the crucial immune-related biomarkers and possible regulatory mechanisms using a combination of weighted gene co-expression network analysis (WGCNA) and ceRNA. Functional enrichment analysis was conducted to detect pathways of immune-related biomarkers in ICH to provide a basis for further mechanism research and immune intervention.

## Materials and methods

### Patients and controls

Seventeen ICH patients were collected from April 2019 to September 2020 in the neurology department of the First Hospital of China Medical University. All ICH patients met the following criteria: (1) first attack, (2) aged between 30 and 70 years, (3) acute onset within 24 h, (4) compliance with the diagnostic standard of the 9th edition of the International Classification of Diseases (ICD9), (5) ICH was confirmed in the basal ganglia using computed tomography (CT), (6) Chinese Han nationality, and (7) no coma. Patients with the following conditions were excluded: (1) secondary ICH-induced by brain tumor, trauma, arteritis, cerebrovascular malformation, and anticoagulant drug use; (2) severe basic diseases such as kidney, liver, heart, blood system, and endocrine system diseases; (3) hemorrhagic transformation; (4) thrombolytic therapy; (5) subarachnoid hemorrhage; and (6) a history of recent trauma and surgery. Ten healthy controls were matched with patients in the ICH group according to age and gender and were randomly selected from the volunteers for a regular health examination in the physical examination center of the First Hospital of China Medical University.

**FIGURE 1:**
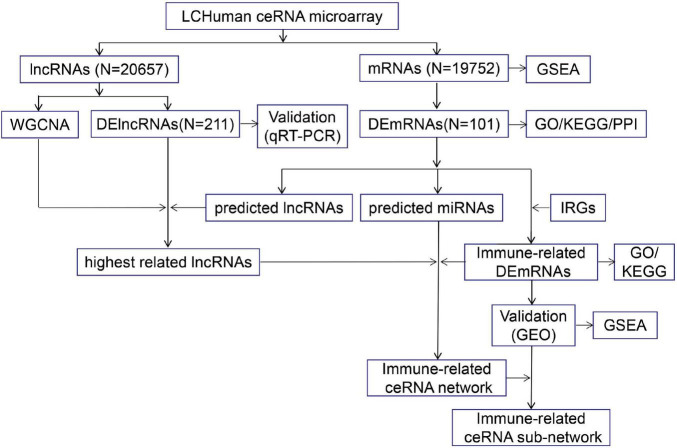
The flow chart of this study. The peripheral blood from ICH patients and control subjects were collected for LCHuman ceRNA microarray and verification using qRT-PCR. The DElncRNAs and DEmRNAs were identified and GO, KEGG, PPI, and GSEA were performed. Then, we got the highest related lncRNAs to ICH by taking the intersection of strongest correlated lncRNAs module by WGCNA, DElncRNAs from our ceRNA microarray, and predicted lncRNAs. We took the intersection of DEmRNAs and IRGs to obtain immune-related DEmRNAs. TargetScan and miRanda were used to predict the interactions between three types of RNAs, and the immune-related ceRNA network was constructed. In addition, we performed GO and KEGG on the immune-related DEmRNAs, verified their expression by the GSE24265 and the GSE125512 from GEO datasets, and conducted GSEA to identify potential pathways of the immune-related DEmRNAs. Finally, we constructed the immune-related ceRNA sub-network.

The study was approved by the ethics committee of the First Hospital of China Medical University (No. 2012-38-1), and all subjects signed an informed consent document. Four cases were randomly selected from the ICH group and control groups, and peripheral blood was collected for LCHuman ceRNA microarray. Peripheral blood of the remaining ICH patients and control subjects was collected for validation by qRT-PCR. The flow chart of this study is shown in Figure 1.

### Peripheral blood collection and original image acquisition

Peripheral blood samples were collected from control subjects and from patients within 24 h after ICH onset. Serum was isolated using a centrifuge at 1,200 rpm for 10 min at 20°C. After that, total RNA was extracted with Trizol (Invitrogen) and purified using QIAGEN RNeasy^®^ Mini Kit (QIAGEN). One-step synthesis of the first and second strands of cDNA were constructed following the manufacturer’s protocol using an RNA Spike-In Kit (Agilent Technologies). Next, cRNA was synthesized and labeled with fluorescent dye cyanine-3-ctp (Cy3). The concentration of cRNA was analyzed by spectrophotometer after purification; high purity cRNA demanded a ratio of A260/A280 close to 2.0 (the reference range 1.9–2.1), which were hybridized with LCHuman ceRNA microarray (Agilent Technologies, 4 × 180 K) at 65°C for 17 h. Finally, the original image was scanned using an Agilent Scanner G5761A (Agilent Technologies) after elution.

### Screening methods of differentially expressed long non-coding ribonucleic acids (DElncRNAs) and mRNAs (DEmRNAs)

The original images and data were processed and extracted using Feature Extraction software (12.0.3.1). Then, we used Genespring software (14.8) for quantile normalization and subsequent processing. The normalized data with at least one group of 100% detected probes were filtered for subsequent analysis. The screening criteria of DElncRNAs and DEmRNAs were a ≥ 2.0 fold change and a *p*-value of < 0.05. Heat map and volcano map were used to visualize the DElncRNAs and DEmRNAs using the ggplot2 package in R.

### Functional enrichment analysis of DEmRNAs

Metascape (Zhou et al., 2019)^1^ was used as a tool for the process and pathway enrichment analysis of DEmRNAs. Terms were grouped into clusters according to similarity with the criteria of a minimum of three count, *P* < 0.01, and an enrichment factor > 1.5. In the hierarchical clustering of enriched terms, Kappa score was used as a similarity measure, and a subtree with a similarity > 0.3 was regarded as a cluster.

### Protein-protein interaction network

Protein-protein interaction (PPI) analysis was conducted in Metascape using the following databases: STRING (Szklarczyk et al., 2019), BioGrid, OmniPath, and InWeb_IM (Stark et al., 2006; Li et al., 2016). When three to 500 proteins were contained in the network, the algorithm of Molecular Complex Detection (MCODE) recognized components of the densely connected network (Bader and Hogue, 2003). Process and enrichment analysis of pathways were used for every MCODE component, respectively.

### Gene set enrichment analysis

Gene set enrichment analysis (GSEA), performed using the clusterProfiler package in R, was applied to clarify significant differences in gene signaling pathways between the ICH and control groups. A pathway term with adjusted *P* < 0.05, FDR < 0.05, and an absolute NSE value of > 1 was considered statistically significant enrichment.

### Identification and functional enrichment analysis of immune-related DEmRNAs

We got immune-related genes (IRGs) from the InnateDB database^2^ with a list of 4,677 genes. Immune-related DEmRNAs in ICH were identified by taking an intersection of IRGs and DEmRNAs, and visualized in a heat map and density plot using the “ggplot2” R package. In addition, the “clusterProfiler” R package was applied to perform GO and KEGG on immune-related DEmRNAs.

### Validation of 10 randomly selected DElncRNAs expression using qRT-polymerase chain reaction

The peripheral blood of ICH patients and controls was collected to validate the expression of ten randomly selected DElncRNAs, namely XIST, FAM182B, LINC00472, LOC101927210, LINC02731, PCBP1-AS1, LINC00174, LOC100131626, HOXB-AS3, and LINC00299. The total RNA of blood samples was isolated using Trizol (Invitrogen). Then, cDNA was synthesized using the HiScript III 1st Strand cDNA Synthesis Kit (+ gDNA wiper) (Vazyme) following the manufacturer’s instructions. qRT-PCR was conducted using ChamQ Universal SYBR qPCR Master Mix (Vazyme) under conditions of 95°C for 30 s, followed by 39 cycles of 95°C for 10 s and 60°C for 30 s in BIO-RAD CFX96TM Real-Time PCR System. GAPDH was selected as a reference gene. Sequences of primers are shown in Supplementary Table 1.

### The analysis of lncRNAs modules using weighted gene co-expression network analysis

WGCNA was used to analyze gene co-expression networks using an R package (Langfelder and Horvath, 2008). In this study, the top 50% ranked lncRNAs by variance analysis were screened, and the sample cluster was used to identify the outlier samples. The appropriate soft threshold was then selected using a function of pickSoftThreshold to construct the network to obtain multiple lncRNAs modules. Two-hundred permutation tests were conducted for preservation statistics to ensure the reliability of the results, and a Z summary score ≤ 2 represented poor preservation. Finally, the correlation analysis of groups and modules were applied to identify the strongest correlated lncRNAs module to ICH.

### Construction of the competing endogenous ribonucleic acids network

The sequence of miRNAs in the ceRNA network was from miRBase (Griffiths-Jones et al., 2006).^3^ TargetScan (Agarwal et al., 2015)^4^ and miRanda (Betel et al., 2008) were used to investigate the interactions between miRNAs-lncRNAs and miRNAs-mRNAs. The intersection of the highly correlated lncRNAs module (green module) by WGCNA with ICH, DElncRNAs from our ceRNA microarray, and predicted lncRNAs, were used for construction of the lncRNAs associated ceRNA network. Cytoscape 3.8.2 (Shannon et al., 2003) was used to visualize the lncRNA-miRNA-mRNA ceRNA network. Moreover, the immune-related lncRNAs-associated ceRNA network of ICH was displayed using a Sankey diagram and visualized using the “ggalluvial” R package.

### Identification of immune-related key biomarkers and pathways in intracerebral hemorrhage

We verified expression of immune-related DEmRNAs in the peripheral blood and brain tissue from ICH patients using the GSE125512 and GSE24265 of Gene Expression Omnibus (GEO) datasets, which were screened using the limma package (Ritchie et al., 2015; Walsh et al., 2019). The intersection of IRGs from the immune database, the DEmRNAs from our ceRNA microarray, and the DEmRNAs from the GSE24265 and GSE125512 datasets are shown using a Venn diagram. Expression of immune-related key biomarkers were exhibited using a violin diagram. Samples were grouped into high-expression and low-expression groups based on median expression of key biomarkers. Single-gene GSEA was performed to detect the possible pathways of immune-related key biomarkers in high and low expression groups using clusterProfiler package in R, with 1,000 × gene set permutations for every analysis; an adjusted *p*-value of < 0.05 and absolute NSE > 1 was considered statistically significant enrichment. A Sankey diagram was used to demonstrate the immune-related ceRNA sub-network. The “ggplot2” R package was used for visualization.

### Statistical analysis

SPSS 25.0 was used to analyze clinical data. *T*-tests were performed to compare measurement data, which were expressed as mean differences ± standard deviation (x ± s), and the Fisher exact probability method was applied to compare count data. A *p*-value of < 0.05 suggested the difference was statistically significant.

## Results

### The clinical characteristics of intracerebral hemorrhage patients and controls

There were seventeen patients in the ICH group with an average age of 55.12 ± 10.98 years, and 10 controls with an average age of 58.00 ± 7.85 years. No significant difference was found in age, history of hypertension, diabetes, smoking, drinking, platelet, hemoglobin, coagulation, or low-density lipoprotein cholesterol (LDL) among the two groups (*P* > 0.05). A higher number of white blood cells in peripheral blood was identified in the ICH group, and the difference was significant statistically (*P* < 0.05) (Table 1).

**TABLE 1:** Comparison of clinical data between ICH group and controls.

Clinical data	Controls (*N* = 10)	ICH group (*N* = 17)	*P*-values
Age(years)	58.00 ± 7.85	55.12 ± 10.98	0.475
Hypertension(%)	40%	70.59%	0.224
Diabetes(%)	30%	35.29%	1.000
Smoking(%)	30%	52.94%	0.424
Drinking(%)	20%	29.41%	0.678
White blood cells(×10^9^/L)	6.63 ± 1.09	8.90 ± 2.38	***0.009***
Platelet(×10^9/L)	226.70 ± 46.97	219.76 ± 66.82	0.776
Hemoglobin(g/L)	156.00 ± 9.52	149.65 ± 14.73	0.235
INR	1.02 ± 0.03	1.00 ± 0.06	0.271
Fibrinogen(g/L)	3.09 ± 0.35	3.15 ± 0.75	0.77
PT(s)	13.02 ± 0.80	13.05 ± 0.67	0.917
APTT(s)	36.51 ± 4.83	34.72 ± 2.80	0.232
LDL(mmol/L)	3.45 ± 0.53	3.00 ± 0.97	0.189

The data in the table were expressed as mean ± standard deviation. The bold and italic value represented p-value of < 0.05.

### Identification of DElncRNAs and DEmRNAs

**FIGURE 2:**
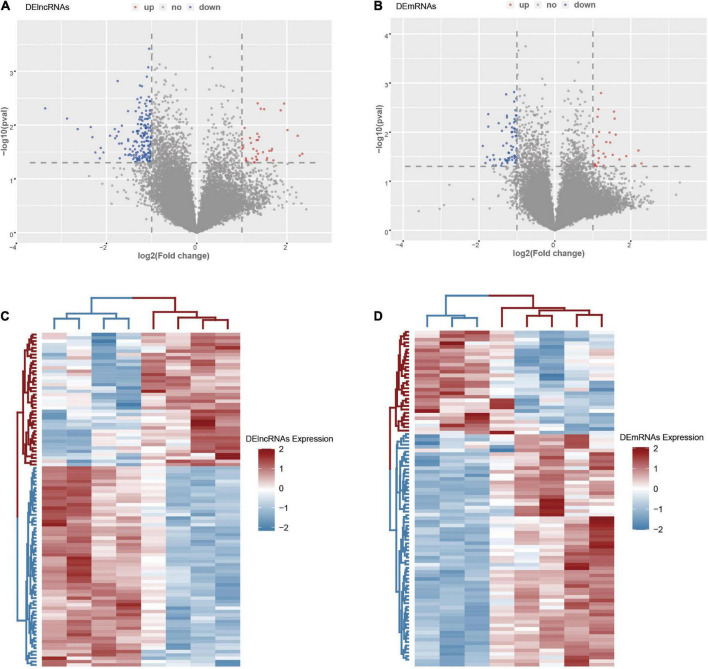
The DElncRNAs and DEmRNAs in the ICH group compared with the control group. **(A)** The volcano map of DElncRNAs in the ICH group compared with the control group. **(B)** The volcano map of DEmRNAs in the ICH group compared with the control group. **(C)** The heat map of the top 100 DElncRNAs in the ICH group compared with the control group. **(D)** The heat map of DEmRNAs in the ICH group compared with the control group. Red represented up-regulation and high expression, while blue represented down-regulation and low expression.

There were 20,657 lncRNAs and 19,752 mRNAs detected in the LCHuman ceRNA microarray of ICH patients compared with controls after normalization of the original data. After screening with the criteria described above, we obtained 211 DElncRNAs (40 up regulation and 171 down regulation) and 101 DEmRNAs (31 up regulation and 70 down regulation). With log_2_FC as abscissa and -log10 (*p*-value) as ordinate, the volcano maps of all DElncRNAs and DEmRNAs are shown, respectively in Figures 2A,B. The heat map of the top 100 DElncRNAs and DEmRNAs are shown in Figures 2C,D.

### Functional enrichment analysis of DEmRNAs in intracerebral hemorrhage

**FIGURE 3:**
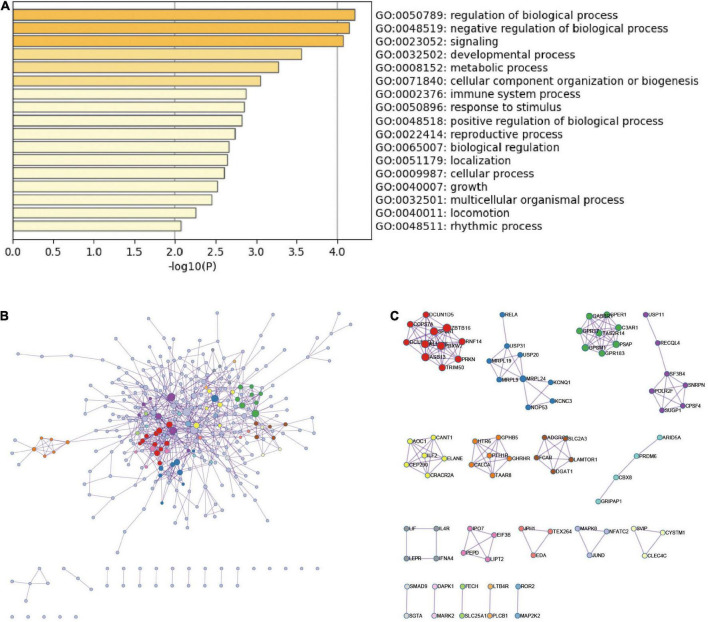
Functional enrichment analysis and PPI network of DEmRNAs in ICH. **(A)** The top-level Gene Ontology biological processes of DEmRNAs in ICH. **(B)** PPI network of DEmRNAs in ICH group and MCODE component in gene table. **(C)** The main protein interaction domains extracted from the protein network.

The top-level GO biological processes are shown in Figure 3A. The bar graph of enriched terms primarily included regulation of biological processes, signaling pathways, metabolic processes, immune system processes, and response to stimulation. The integration results of the top 20 most enriched pathways, Hallmark gene set, KEGG pathway, and GO biological process according to the lowest *p*-value are shown in Supplementary Figures 1A,B and Supplementary Table 2. The results were mainly as follows: Wnt signaling pathway regulation, regulation of proteasome ubiquitin dependent protein catabolism, negative regulation of CREB transcription factor activity, PID CD8 TCR downstream pathway regulation, mitochondrial autophagy, and myeloid leukocyte activation.

### Protein-protein interaction enrichment analysis of DEmRNAs in intracerebral hemorrhage

According to the DEmRNAs in the peripheral blood of ICH patients, the MCODE algorithm in metascape was used to extract the physical interaction between corresponding proteins, namely PPI, from the database to form a network (Figures 3B,C). The specific terms of MCODE are shown in Supplementary Table 3. The enriched terms of PPI mainly included antigen processing, Class I MHC mediated antigen processing and presentation, mitochondrial translation termination, ADORA2B-mediated anti-inflammatory cytokines production, neutrophil activation involved in immune response, and JAK-STAT signaling pathway.

### Gene-related signaling pathways based on gene set enrichment analysis in intracerebral hemorrhage

**FIGURE 4:**
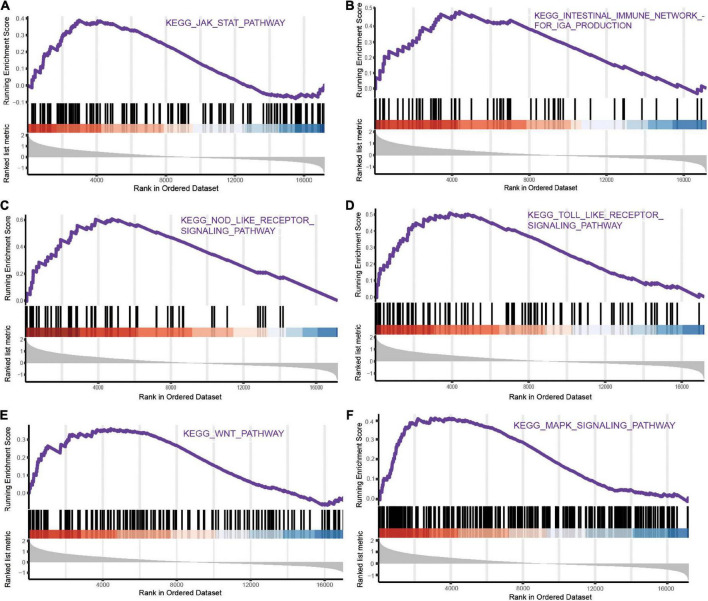
The representative pathways with significant differences of mRNAs in ICH based on GSEA. **(A)** JAK-STAT signaling pathway. **(B)** Intestinal immune network pathway. **(C)** NOD-like receptor signaling pathway. **(D)** Toll-like receptor signaling pathway. **(E)** Wnt signaling pathway. **(F)** MAPK signaling pathway.

We performed GSEA and discovered representative signaling pathways with significant differences in the intestinal immune network pathway, JAK-STAT signaling pathway, MAPK signaling pathway, Toll-like receptor signaling pathway, NOD-like receptor signaling pathway, and Wnt signaling pathway (Figure 4 and Supplementary Table 4).

### Identification and functional enrichment analysis of immune-related DEmRNAs in intracerebral hemorrhage

**FIGURE 5:**
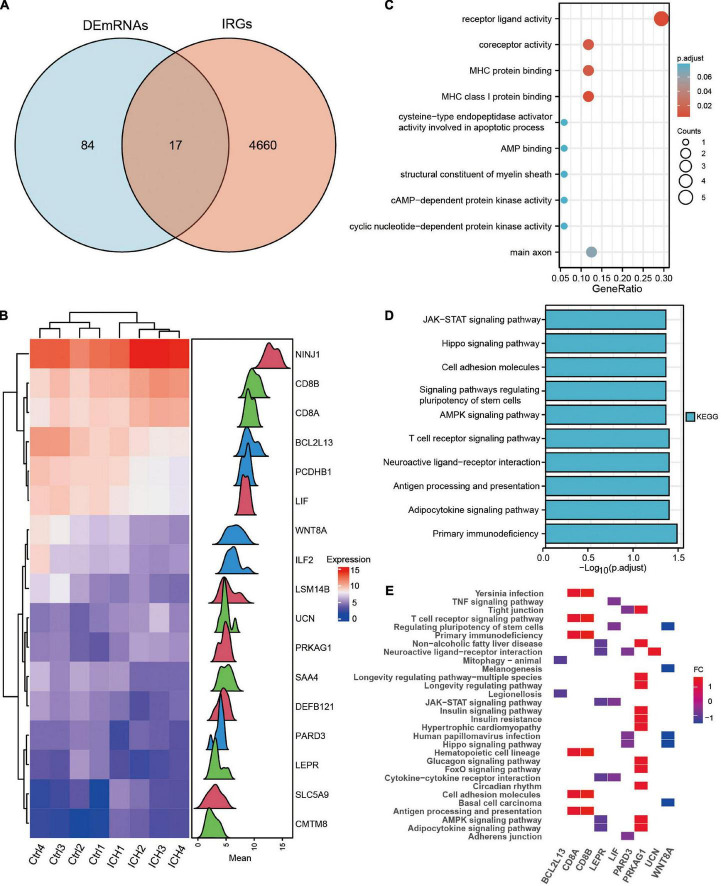
Identification and functional enrichment analysis of the immune-related DEmRNAs in ICH. **(A)** The venn diagram showed the intersection genes of DEmRNAs and IRGs in ICH. **(B)** The heatmap and density plot of the 17 immune-related DEmRNAs in ICH. **(C)** The top 10 Gene Ontology biological processes of immune-related DEmRNAs in ICH. **(D)** The top 10 KEGG enrichment analysis of immune-related DEmRNAs in ICH. **(E)** The heatmap of KEGG pathways of immune-related DEmRNAs in ICH.

The Venn diagram showed 17 immune-related DEmRNAs by taking the intersection of 101 DEmRNAs and 4,677 IRGs in ICH patients (Figure 5A and Supplementary Table 5). The heat map and density plot show the expression of the above 17 immune-related DEmRNAs (Figure 5B). GO and KEGG of immune-related DEmRNAs showed that the antigen processing and presentation, Hippo signaling pathway, JAK-STAT signaling pathway, and other pathways were significantly enriched in ICH (Figures 5C–E).

### Validation of 10 randomly selected DElncRNAs expressed in intracerebral hemorrhage

**FIGURE 6:**
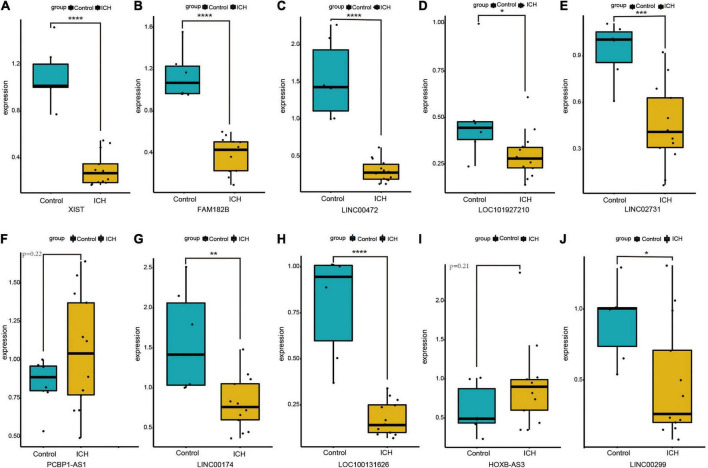
The expression of ten DElncRNAs in peripheral blood of ICH patients compared with control subjects using qRT-PCR. **(A)** The qRT-PCR results of XIST. **(B)** The qRT-PCR results of FAM182B. **(C)** The qRT-PCR results of LINC00472. **(D)** The qRT-PCR results of LOC101927210. **(E)** The qRT-PCR results of LINC02731. **(F)** The qRT-PCR results of PCBP1-AS1. **(G)** The qRT-PCR results of LINC00174. **(H)** The qRT-PCR results of LOC100131626. **(I)** The qRT-PCR results of HOXB-AS3. **(J)** The qRT-PCR results of LINC00299. *P < 0.05, **P < 0.01, ***P < 0.001.

We randomly selected 10 DElncRNAs and validated their expression in the peripheral blood of ICH patients and controls using qRT-PCR. The results showed that XIST, FAM182B, LINC00472, LOC101927210, LINC02731, LINC00174, LOC100131626, and LINC00299 were significantly down-regulated in the ICH group. In addition, the expression of PCBP1-AS1 and HOXB-AS3 showed an up-regulation trend in the ICH patients compared with the control subjects, although the difference was not significant statistically. The expression of 10 DElncRNAs identified using qRT-PCR are displayed in Figure 6.

### Analysis of long non-coding ribonucleic acids modules in intracerebral hemorrhage using weighted gene co-expression network analysis

**FIGURE 7:**
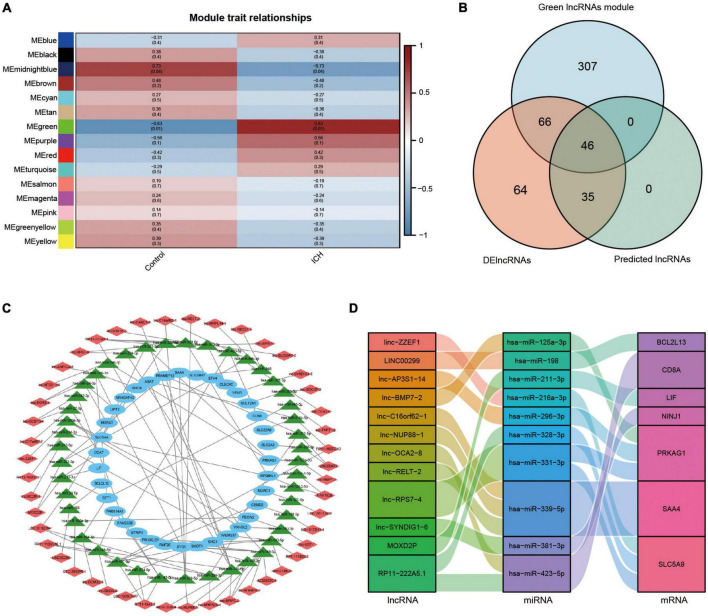
Analysis of lncRNAs modules using WGCNA and the construction of lncRNAs associated ceRNA network in ICH. **(A)** Module trait relationships between ICH patients and control subjects. Red represented positive correlation, blue represented negative correlation, and darker colors represented higher correlation. **(B)** The venn diagram showed the intersection of the green lncRNAs module by WGCNA, the DElncRNAs from our microarray, and the predicted lncRNAs. **(C)** The constructed ceRNA network of lncRNAs from the intersection, the predicted miRNAs, and the DEmRNAs from our microarray was visualized by Cytoscape. Red diamond represented lncRNAs, green triangle represented miRNAs, and blue ellipse represented mRNAs. **(D)** The Sankey diagram showed the immune-related lncRNA-miRNA-mRNA ceRNA network in ICH.

We firstly analyzed the top 50% of ranked lncRNAs modules using WGCNA. The cluster sample dendrogram showed no outlier samples (Supplementary Figure 2A), and 18 was chosen as the appropriate soft threshold selected by the pickSoftThreshold function (Supplementary Figure 2B). Co-expression network was conducted; 16 lncRNAs modules are shown in different colors, with the biggest module being turquoise (3,899 lncRNAs) and smallest module midnight blue (38 lncRNAs) (Supplementary Figure 2C). Preservation statistics were applied to evaluate the stability of the above lncRNAs modules, and the black and gold modules with a Z summary score of < 2 were removed. Preservation median rank and preservation Z summary score of the remained preserved lncRNAs modules are shown in Supplementary Figure 2D. Finally, we conducted the correlation analysis and discovered that the strongest relationship was between the green lncRNAs module and ICH (*r* = 0.83, *p* = 0.01). The results of module trait relationships are shown in Figure 7A.

### Construction of long non-coding ribonucleic acids associated competing endogenous ribonucleic acid network in intracerebral hemorrhage

We constructed a ceRNA network of lncRNA-miRNA-mRNA to elucidate the regulation between the three types of RNAs in ICH. We took the intersection of the green lncRNAs module (419 lncRNAs) most strongly correlated with ICH by WGCNA, 211 significant DElncRNAs from our ceRNA microarray, and 81 predicted lncRNAs by the above software to construct a network of lncRNAs associated ceRNA (Figure 7B). The lncRNA-miRNA-mRNA ceRNA network was created and visualized using Cytoscape (Figure 7C). The network contained 46 lncRNAs, 44 miRNAs, and 35 mRNAs. Moreover, the immune-related ceRNA network was constructed for ICH and was visualized using a Sankey diagram with 12 lncRNAs, 10 miRNAs, and 7 mRNAs (Figure 7D).

### Identification of immune-related key biomarkers and pathways in the peripheral blood and brain tissue of intracerebral hemorrhage patients

**FIGURE 8:**
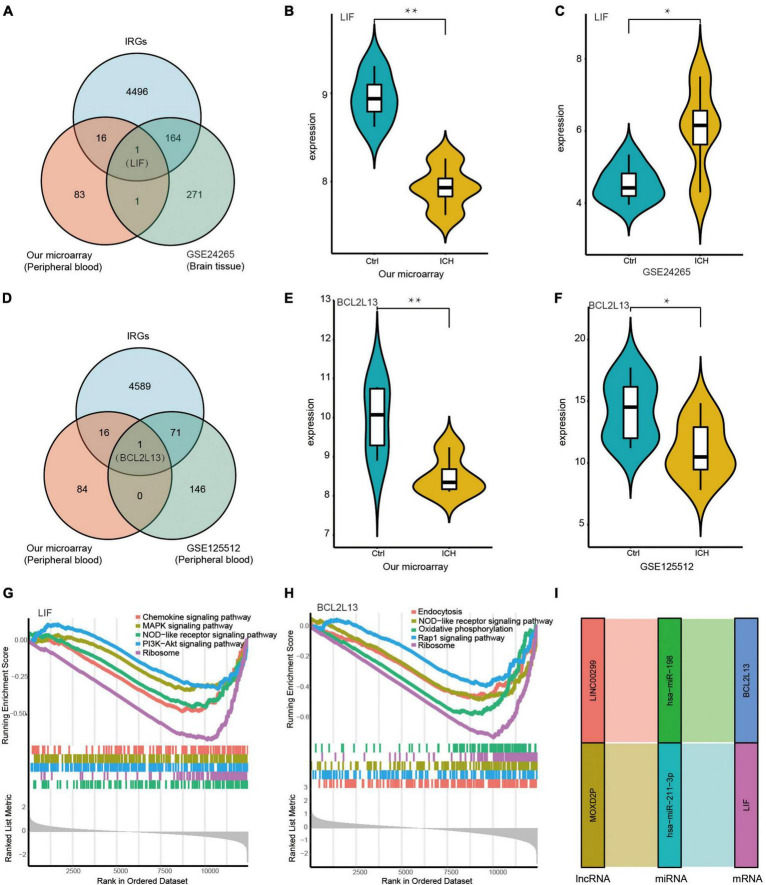
Identification of key biomarkers and pathways of the immune-related DEmRNAs in the peripheral blood and the brain tissue from ICH patients. **(A)** The venn diagram showed LIF was at the intersection of the IRGs from immune database, the DEmRNAs in peripheral blood from our microarray, and the DEmRNAs in brain tissue from the GSE24265 dataset of ICH patients. **(B)** The expression of LIF in the peripheral blood from our ceRNA microarray. **(C)** The expression of LIF in the brain tissue from the GSE24265 dataset of ICH patients. **(D)** The venn diagram showed BCL2L13 was at the intersection of the IRGs from immune database, the DEmRNAs in the peripheral blood from our microarray, and the DEmRNAs in the peripheral blood from the GSE125512 dataset of ICH patients. **(E)** The expression of BCL2L13 in the peripheral blood from our ceRNA microarray. **(F)** The expression of BCL2L13 in the peripheral blood from the GSE125512 dataset of ICH patients. **(G)** The five representative results of GSEA for LIF. **(H)** The five representative results of GSEA for BCL2L13. **(I)** The immune-related ceRNA sub-network of LIF and BCL2L13, including MOXD2P-miR-211-3p-LIF and LINC00299-miR-198-BCL2L13. *P < 0.05, **P < 0.01.

Leukaemia inhibitory factor (LIF) was at the intersection of IRGs from the immune database, DEmRNAs in peripheral blood from our microarray, and DEmRNAs in the brain tissue from the GSE24265 dataset of ICH patients (Figure 8A). The expression of LIF was low in the peripheral blood from our ceRNA microarray, but was high in the brain tissue from the GSE24265 dataset in ICH patients compared with controls (Figures 8B,C). In addition, BCL2L13 was at the intersection of IRGs from the immune database, DEmRNAs in the peripheral blood from our microarray, and DEmRNAs in the peripheral blood from the GSE125512 dataset of ICH patients (Figure 8D). The expression of BCL2L13 was low not only in the peripheral blood from our ceRNA microarray, but also in the GSE125512 dataset from ICH patients in comparison with controls (Figures 8E,F). The representative results of GSEA for LIF included the PI3K-Akt signaling pathway, NOD-like receptor signaling pathway, MAPK signaling pathway, and others (Figure 8G). The representative results of GSEA for BCL2L13 were as follows: oxidative phosphorylation, Rap1 signaling pathway, and NOD-like receptor signaling pathway (Figure 8H). In addition, the Sankey diagram showed the immune-related ceRNA sub-network of LIF and BCL2L13, including MOXD2P-miR-211-3p-LIF and LINC00299-miR-198-BCL2L13 (Figure 8I).

## Discussion

In the present study, we identified the differing peripheral blood expressions of DElncRNAs and DEmRNAs in ICH patients compared with controls using a LCHuman ceRNA microarray. We then performed GO enrichment, KEGG pathway, PPI analysis, and GSEA, and discovered that the immune system process was an important part of the pathological process of ICH, which had been partially confirmed in our previous research (Xi et al., 2017, 2018; Hu et al., 2019, 2020; Lu et al., 2020; Zhang et al., 2020). Additionally, we found that the number of white blood cells increased significantly in the peripheral blood of the ICH group compared with the control group, which suggested that immune and inflammation responses participated in the secondary injury following ICH.

To identify immune-related biomarkers in ICH, we obtained the immune-related DEmRNAs through the InnateDB database, and found that the Hippo signaling pathway, JAK-STAT signaling pathway, and other pathways were significantly enriched using functional enrichment analysis. The Hippo signaling pathway regulates the tissue regeneration and development, which are associated with inflammation, oxidative stress, and cell death. Zhang et al discovered that the Hippo signaling pathway was activated to promote neuronal cell death and the inflammatory response after ICH in rats (Zhang et al., 2019). The specific mechanism of pathway activation and the changes of molecular expression involved remain unknown, and therefore require further study. The JAK-STAT signaling pathway is involved in tumor formation, inflammation, and hormone secretion in the brain (Nicolas et al., 2013). Activation of the JAK-STAT signaling pathway following ICH has not been previously reported. Our results provided a foundation for the study of immune-related pathways in ICH.

To confirm the reliability of our ceRNA microarray, 10 DElncRNAs were randomly selected for qRT-PCR. The results showed a significantly different expression of XIST, FAM182B, LINC00472, LOC101927210, LINC02731, LINC00174, LOC100131626, and LINC00299 in the peripheral blood of ICH patients compared with controls, which was consistent with our microarray. PCBP1-AS1 and HOXB-AS3 were up-regulated in the ICH group, the expression trend of which were the same as in our microarray, although the difference was not significant statistically on account of the small sample size or primer design. In subsequent experiments, we will include more samples for qRT-PCR, and adjust the scheme of primer design for verification.

In addition, we verified the expression of immune-related DEmRNAs from GEO datasets and noticed a significantly different expression in LIF and BCL2L13 from the brain tissue and in peripheral blood of ICH patients, respectively, compared with controls. It was noteworthy that only two immune-related DEmRNAs were present in the intersection of our microarray and GEO datasets. We analyzed the reasons as follows. Firstly, the samples and collection time for ICH and controls were different; peripheral blood samples were collected in patients within 24 h after ICH onset and from healthy controls in our microarray. In the GSE125512 dataset, gene expression in the peripheral blood was compared between 24 h and 96 (± 6) h after the onset of ICH. In the GSE24265 dataset, brain samples from perihematomal tissue and the corresponding contralateral areas in ICH patients were compared within 5 h after death. Secondly, the screening criteria of DEmRNAs differed; our screening criteria was stricter with a ≥ 2.0 fold change and a *p*-value of < 0.05, while for GSE125512 with FDR < 0.1, and for GSE24265 with a > 1.3 fold change and a *p*-value of < 0.05. The above differences may have led to only LIF and BCL2L13 present in the intersection of our microarray and GEO datasets. The results suggested that LIF was significantly differentially expressed not only in the peripheral blood but also in the brain tissue. In addition, BCL2L13 showed a significantly lower expression in the early stage (24 h) of ICH.

LIF belongs to the IL-6 cytokine family, and is described as a glycoprotein that inhibits M1 myeloid leukemia cell proliferation and induces differentiation. LIF also acts on many cell types and is categorized as a cholinergic differentiation factor, which possesses extensive functions in development and maturation of the nervous system (Suzuki et al., 2000). It was reported that LIF induced an astrocyte progenitor cell line to differentiate into mature astrocytes (Yoshida et al., 1993). Astrocytes regulate ion homeostasis, synapse formation, and neurovascular regeneration in the nervous system, and affect the inflammatory response together with microglia in states of injury (Khakh and Sofroniew, 2015). In addition, astrocytes produce a variety of cytokines, which stimulate microglia and gathered immune cells to migrate to nervous system (Mayo et al., 2014). Innate immune cells cause neuroinflammation and neuronal death by producing oxidative stress, chemokines, and proinflammatory cytokines (Lee et al., 2010; Noubade et al., 2014). In our study, LIF was in specific terms of MCODE 9 by PPI analysis, the description of which was JAK-STAT signaling pathway. The above result suggested that LIF might exert its function through the JAK-STAT signaling pathway in ICH. Studies have demonstrated that LIF, together with its receptors gp130 and LIFRβ, form a signal transduction complex, which catalyze JAK1, initiate tyrosine phosphorylation cascade, and activate the JAK-STAT signaling pathway (Skiniotis et al., 2008; Nicola and Babon, 2015). Immune regulation, apoptosis, cell proliferation, and differentiation are involved in the biological processes of the JAK-STAT signaling pathway (Bolli et al., 2003). JAK-STAT is the main signaling pathway effected by cytokines, which is of great significance in inflammatory and autoimmune diseases (O’Shea et al., 2015; Xin et al., 2020). STAT3 is important in differentiation of T-helper (Th) 17 cells (Abroun et al., 2015). IL-17, secreted by Th17 cells, promotes an extracellular immune response, and IL-22 maintains barrier integrity (Wolk et al., 2004; Conti et al., 2015). Activation of the STAT3 signaling pathway is significant in reactive astrocyte proliferation. Research has also reported that LIF can act on reactive astrocyte proliferation by activating the STAT3 signaling pathway in a rat ICH model (Zhou et al., 2017), which verifies our results *in vivo*. Moreover, the PI3K-Akt and MAPK signaling pathways were representative results of single-gene GSEA for LIF in this study. Previous studies demonstrated that LIF not only induced STAT3 acetylation by PI3K-Akt activation, but also activated the MAPK-dependent signaling pathway in some cell lines, which were identical to our research (Ohbayashi et al., 2007; Suman et al., 2013). The above results need to be further verified in cell and animal models of ICH.

B cell lymphoma 2 (BCL2) family proteins, comprised of pro-apoptotic or anti-apoptotic proteins, take over the permeabilization of mitochondrial outer membranes and regulate cell death (Meng et al., 2021). BCL2-like 13 (BCL2L13), apoptosis facilitator on human chromosome 22, promotes fragmentation and autophagy of mitochondrial and regulates apoptosis. The physiological roles of BCL2L13 include regulation of growth, development, and energy metabolism, while the pathological implications include cancer, cardiovascular disease, and degenerative disease (Otsu et al., 2015). Autophagy regulates the immune system by removing dysfunctional mitochondria. Moreover, autophagy of mitochondria may limit the secretion of inflammatory cytokines and regulate the process of antigen presentation and homeostasis of immunocytes. Studies have elucidated that mitophagy controls the intracellular anti-inflammatory mechanism, possibly by inhibiting the production of IL1B and IL18, which act on NLR family pyrin domain-containing 3 (NLRP3) and IL1B by degrading lysosomes (Shi et al., 2012; Zhong et al., 2016). Mitophagy affects the differentiation, activation, development and death of immunocytes such as T cells, natural killer cells, and macrophages (Salio et al., 2014). BCL2L13 can raise oxidative phosphorylation, inhibit apoptosis, and regulate autophagy of mitochondria. Knockdown of BCL2L13 results in a significant decrease in oxidative phosphorylation and enhances energy produced by glycolysis in mesenchymal stem cells (Fujiwara et al., 2019). The above results disclose that BCL2L13 is closely related to oxidative phosphorylation, consistent with the results of single-gene GSEA for BCL2L13 in our study.

The lncRNA-miRNA-mRNA ceRNA regulation network has attracted increasing attention as a potential regulatory mechanism of disease. Until now, there were reports on nervous system diseases such as cerebral infarction, glioblastoma, Alzheimer’s disease (AD), and peripheral nerve injury (Qian et al., 2018; Zou et al., 2019; Liu et al., 2020; Ma et al., 2020). WGCNA is widely used to discover co-expression gene modules which are related to clinical features. In order to further explore the regulatory mechanism underlying the process of ICH, we performed WGCNA and obtained the green module with the strongest relation of lncRNAs to ICH. On the basis of the WGCNA results, we established immune-related lncRNA-miRNA-mRNA networks using ceRNA theory. In ceRNA networks, lncRNAs sponge miRNAs to interact with mRNAs and regulate their expression (Guo et al., 2015). Based on the above theory, lncRNAs act as pivotal regulators of ceRNA networks in ICH. Thus, we sought the regulation axes and correlated lncRNAs with LIF and BCL2L13 in our constructed immune-related ceRNA network, including MOXD2P-miR-211-3p-LIF and LINC00299-miR-198-BCL2L13. MOXD2 encode a monooxygenase dopamine β-hydroxylase (DBH)-like 2 proteins, which play a role in the metabolism of neurotransmitters (Goh et al., 2016) and are related to neuropsychiatric diseases (Timmers et al., 2004; Cubells et al., 2011). For example, there is an interaction between DBH and the polymorphisms of proinflammatory cytokine IL6 and IL1A, resulting in the susceptibility of AD (Combarros et al., 2010). DBH is related to cognitive function, but the neurocognitive function of patients with DBH deficiency is not affected because of neurotransmitter compensatory mechanisms (Jepma et al., 2011). However, the correlation between MOXD2P and ICH remains poorly understood. We inferred the possible mechanism of MOXD2P-mediated regulation of LIF in ICH was sponging miR-211-3p from the perspective of ceRNA theory. The down-regulation of MOXD2P and LIF in peripheral blood might be related to the inflammatory response of ICH. Additionally, LINC00299, located on chromosome 2, is abundantly expressed in the brain and related to neurodevelopmental disabilities (Talkowski et al., 2012). In atherosclerosis, the expression of LINC00299 is upregulated, promoting migration and proliferation, and alleviating apoptosis of vascular smooth muscle cells by sponging miRNAs (Liu et al., 2019; Chang et al., 2022). LINC00299 regulated the expression of BCL2L13 in the ceRNA network, which may be involved in the pathological immune process of ICH. The precise character and mechanism of LINC00299 in ICH requires further investigation. In our constructed immune-related ceRNA sub-network, MOXD2P-miR-211-3p-LIF and LINC00299-miR-198-BCL2L13 axes were identified and speculated to have significant roles in the immune mechanism of ICH. Our next experiments will explore the possible immune-related regulatory mechanism of the above axes in the pathological process of ICH.

In this study, we constructed immune-related lncRNA-miRNA-mRNA ceRNA networks that were highly related to ICH by combining WGCNA and ceRNA in order to uncover immune-related molecules and potential regulatory mechanism in the pathological process of ICH. The results of our study could lay a foundation for further study of pathological immune mechanisms, and provide new targets of immune intervention for ICH from the perspective of ceRNA.

However, there were few limitations in our study. Firstly, the sample size in the study was relatively small, despite the fact that we verified the expression of ten randomly selected DElncRNAs using qRT-PCR and immune-related DEmRNAs with GEO datasets. Two in ten randomly selected lncRNAs had the same expression trend with our microarray; however, there was no statistically significant difference, so a larger sample size or an adjusted primer design scheme may be needed for verification in subsequent experiments. Additionally, the samples and collection time in the ICH and control groups from the GSE24265 and GSE125512 datasets were different from our microarray, which might reduce the efficiency of verification. We require more samples, including the peripheral blood and the brain tissue, for subsequent verification. Secondly, although we identified immune-related biomarkers and possible pathways of ICH by comprehensive analysis of WGCNA, ceRNA, and functional enrichment analysis, its specific roles and precise regulatory mechanism in ICH were not explicit. The interactions between three types of RNAs, functional experiments, and immune-related pathways require further verification *in vivo* and *in vitro*, which would be the core of our further experiments.

## Conclusion

In summary, our study was the first to construct an immune-related ceRNA network derived from WGCNA, and to identify LIF and BCL2L13 as pivotal immune-related biomarkers from the peripheral blood of ICH patients. MOXD2P-miR-211-3p-LIF and LINC00299-miR-198-BCL2L13 axes were indicated in the immune regulatory mechanism of ICH. The goal of our study was to offer innovative insights into the underlying regulation mechanism and possible immune intervention targets for ICH.

## Data availability statement

The datasets presented in this study can be found in online repositories. The names of the repository/repositories and accession number(s) can be found in the article/Supplementary material, further inquiries can be directed to the corresponding authors.

## Ethics statement

The studies involving human participants were reviewed and approved by the First Hospital of China Medical University. The patients/participants provided their written informed consent to participate in this study.

## Author contributions

ZH and HZ designed the study and revised the manuscript. YH, YW, FJ, and LT collected the peripheral blood and clinical data. YH, XX, and WZ analyzed the data. YH wrote the manuscript. All authors have read and approved the final manuscript.

## Footnotes

1
http://metascape.org

2
http://www.innatedb.com

3
http://www.mirbase.org/

4
http://www.targetscan.org/
