# Supplementary material for: Comprehensive analysis of immune-related biomarkers and pathways in intracerebral hemorrhage using weighted gene co-expression network analysis and competing endogenous ribonucleic acid
Source: Front Mol Neurosci. 2022 Sep 26;15:955818. doi: 10.3389/fnmol.2022.955818 (PMC9549172; doi:10.3389/fnmol.2022.955818)
Supplement: Supplementary file 1 [file Data_Sheet_1.docx]

Table S1 Primer sequences of ten randomly selected DElncRNAs for qRT-PCR.

| Gene name | Primer sequence（5’-3’） | Base number |
| --- | --- | --- |
| XIST-F | TCTAGTCCCCCAACACCCTT | 20 |
| XIST-R | GGAGGACGTGTCAAGAAGACA | 21 |
| FAM182B-F | TTGGATGTGGAACGTGGAGG | 20 |
| FAM182B-R | CGCAACTGTAGGCGTTTCTG | 20 |
| LINC00472-F | TTTCTCGACTCGTCGTCAGC | 20 |
| LINC00472-R | CAACATGTCTGGTGCCGC | 18 |
| LOC101927210-F | TCACAGGCACCTTCCATGAC | 20 |
| LOC101927210-R | AGCACAGGTGACACTGATGG | 20 |
| LINC02731-F | GGTGCTCATACACACAGCCT | 20 |
| LINC02731-R | TGGCCTTTGTGCTTAGGGAG | 20 |
| PCBP1-AS1-F | CTGGGATTACAGGCGTGAG | 19 |
| PCBP1-AS1-R | TAGGGAAGAGGAGAACAGATTA | 22 |
| LINC00174-F | CAAACAGAAGGCAAATAAAC | 20 |
| LINC00174-R | TAGCGATAGTCAAGTAAACC | 20 |
| LOC100131626-F | CAACCCATCACCTAGCCT | 18 |
| LOC100131626-R | GACATCAGCAGATTCCCT | 18 |
| HOXB-AS3-F | AAGTAGAGCCTCCACGACCCT | 21 |
| HOXB-AS3-R | TGGTGCGGATATCGCTGGG | 19 |
| LINC00299-F | GAATAAATCTGAAGGCCCATCAC | 23 |
| LINC00299-R | CAAGGTTCTCATAAGGTTACAGG | 23 |
| GAPDH-F | GAACGGGAAGCTCACTGG | 18 |
| GAPDH-R | GCCTGCTTCACCACCTTCT | 19 |

Table S2 Top 20 clusters with representative enriched terms of DEmRNAs in ICH.

| Term | Category | Description | Count | % | Log10(P) | Log10(q) |
| --- | --- | --- | --- | --- | --- | --- |
| hsa04310 | KEGG Pathway | Wnt signaling pathway | 13 | 2.37 | -4.53 | -0.4 |
| GO:0032434 | GO Biological Processes | regulation of proteasomal ubiquitin-dependent protein catabolic process | 11 | 2.01 | -4.21 | -0.4 |
| GO:0032792 | GO Biological Processes | negative regulation of CREB transcription factor activity | 3 | 0.55 | -4.15 | -0.4 |
| GO:1905114 | GO Biological Processes | cell surface receptor signaling pathway involved in cell-cell signaling | 27 | 4.93 | -4.07 | -0.4 |
| M272 | Canonical Pathways | PID CD8 TCR DOWNSTREAM PATHWAY | 7 | 1.28 | -3.57 | -0.18 |
| ko04137 | KEGG Pathway | Mitophagy - animal | 7 | 1.28 | -3.57 | -0.18 |
| GO:0002076 | GO Biological Processes | osteoblast development | 4 | 0.73 | -3.56 | -0.18 |
| R-HSA-500792 | Reactome Gene Sets | GPCR ligand binding | 21 | 3.83 | -3.42 | -0.18 |
| GO:0006488 | GO Biological Processes | dolichol-linked oligosaccharide biosynthetic process | 4 | 0.73 | -3.27 | -0.07 |
| GO:1901615 | GO Biological Processes | organic hydroxy compound metabolic process | 23 | 4.2 | -3.25 | -0.07 |
| GO:0099536 | GO Biological Processes | synaptic signaling | 27 | 4.93 | -3.17 | -0.07 |
| GO:0016575 | GO Biological Processes | histone deacetylation | 7 | 1.28 | -3.05 | -0.02 |
| hsa04071 | KEGG Pathway | Sphingolipid signaling pathway | 9 | 1.64 | -3.03 | -0.02 |
| GO:0002274 | GO Biological Processes | myeloid leukocyte activation | 25 | 4.56 | -2.87 | -0.01 |
| GO:0071872 | GO Biological Processes | cellular response to epinephrine stimulus | 3 | 0.55 | -2.85 | -0.01 |
| GO:1903020 | GO Biological Processes | positive regulation of glycoprotein metabolic process | 4 | 0.73 | -2.82 | -0.01 |
| GO:0009416 | GO Biological Processes | response to light stimulus | 15 | 2.74 | -2.78 | -0.01 |
| GO:0031667 | GO Biological Processes | response to nutrient levels | 20 | 3.65 | -2.78 | -0.01 |
| M60 | Canonical Pathways | PID NFAT TFPATHWAY | 5 | 0.91 | -2.75 | -0.01 |
| GO:2000344 | GO Biological Processes | positive regulation of acrosome reaction | 3 | 0.55 | -2.74 | -0.01 |

Table S3 The specific terms of MCODE in PPI enrichment analysis of DEmRNAs in ICH.

| MCODE | GO | Description | Log10(P) |
| --- | --- | --- | --- |
| MCODE_1 | R-HSA-8951664 | Neddylation | -14.5 |
| MCODE_1 | R-HSA-983168 | Antigen processing: Ubiquitination & Proteasome degradation | -13.5 |
| MCODE_1 | R-HSA-983169 | Class I MHC mediated antigen processing & presentation | -12.9 |
| MCODE_2 | CORUM:324 | 39S ribosomal subunit, mitochondrial | -6.4 |
| MCODE_2 | CORUM:320 | 55S ribosome, mitochondrial | -5.8 |
| MCODE_2 | R-HSA-5419276 | Mitochondrial translation termination | -5.6 |
| MCODE_3 | R-HSA-418594 | G alpha (i) signalling events | -14.8 |
| MCODE_3 | R-HSA-500792 | GPCR ligand binding | -11.6 |
| MCODE_3 | R-HSA-373076 | Class A/1 (Rhodopsin-like receptors) | -7.9 |
| MCODE_4 | R-HSA-72163 | mRNA Splicing - Major Pathway | -9.6 |
| MCODE_4 | R-HSA-72172 | mRNA Splicing | -9.5 |
| MCODE_4 | R-HSA-72203 | Processing of Capped Intron-Containing Pre-mRNA | -9 |
| MCODE_5 | R-HSA-9660821 | ADORA2B mediated anti-inflammatory cytokines production | -14 |
| MCODE_5 | R-HSA-9664433 | Leishmania parasite growth and survival | -13.4 |
| MCODE_5 | R-HSA-9662851 | Anti-inflammatory response favouring Leishmania parasite infection | -13.4 |
| MCODE_6 | R-HSA-6798695 | Neutrophil degranulation | -10.6 |
| MCODE_6 | GO:0043312 | neutrophil degranulation | -10.6 |
| MCODE_6 | GO:0002283 | neutrophil activation involved in immune response | -10.6 |
| MCODE_7 | R-HSA-6798695 | Neutrophil degranulation | -8.8 |
| MCODE_7 | GO:0043312 | neutrophil degranulation | -8.8 |
| MCODE_7 | GO:0002283 | neutrophil activation involved in immune response | -8.8 |
| MCODE_9 | ko04630 | Jak-STAT signaling pathway | -9 |
| MCODE_9 | hsa04630 | JAK-STAT signaling pathway | -8.9 |
| MCODE_9 | ko04060 | Cytokine-cytokine receptor interaction | -8.1 |
| MCODE_11 | R-HSA-6798695 | Neutrophil degranulation | -5.3 |
| MCODE_11 | GO:0043312 | neutrophil degranulation | -5.3 |
| MCODE_11 | GO:0002283 | neutrophil activation involved in immune response | -5.3 |
| MCODE_12 | ko04380 | Osteoclast differentiation | -7 |
| MCODE_12 | hsa04380 | Osteoclast differentiation | -6.9 |

Table S4 Representative signaling pathways related to ICH identified by GSEA.

| ID | set Size | ES | NES | p.adjust | q values |
| --- | --- | --- | --- | --- | --- |
| KEGG_JAK_STAT_SIGNALING_PATHWAY | 91 | 0.49 | 1.60 | 0.001 | 0.001 |
| KEGG_INTESTINAL_IMMUNE_NETWORK_FOR_IGA_PRODUCTION | 37 | 0.56 | 1.67 | 0.002 | 0.003 |
| KEGG_NOD_LIKE_RECEPTOR_SIGNALING_PATHWAY | 44 | 0.63 | 1.91 | 0.000 | 0.001 |
| KEGG_TOLL_LIKE_RECEPTOR_SIGNALING_PATHWAY | 70 | 0.55 | 1.77 | 0.000 | 0.001 |
| KEGG_WNT_SIGNALING_PATHWAY | 97 | 0.41 | 1.36 | 0.029 | 0.031 |
| KEGG_MAPK_SIGNALING_PATHWAY | 201 | 0.47 | 1.63 | 0.000 | 0.000 |

Table S5 The expression of 17 immune-related DEmRNAs in ICH

| Gene Symbol | Gene Name | TargetID | Chr | log2FC | regulation | pval |
| --- | --- | --- | --- | --- | --- | --- |
| LIF | leukemia inhibitory factor | NM_002309 | chr22 | -1.02 | down | 0.002 |
| CMTM8 | CKLF-like MARVEL transmembrane domain containing 8 | NM_178868 | chr3 | 1.56 | up | 0.004 |
| PCDHB1 | protocadherin beta 1 | NM_013340 | chr5 | -1.05 | down | 0.009 |
| DEFB121 | defensin, beta 121 | NM_001171832 | chr20 | -1.39 | down | 0.009 |
| CD8B | CD8b molecule | NM_004931 | chr2 | 1.35 | up | 0.016 |
| WNT8A | wingless-type MMTV integration site family, member 8A | NM_058244 | chr5 | -1.90 | down | 0.019 |
| NINJ1 | ninjurin 1 | NM_004148 | chr9 | 1.52 | up | 0.029 |
| CD8A | CD8a molecule | NM_001768 | chr2 | 1.01 | up | 0.030 |
| BCL2L13 | BCL2-like 13 (apoptosis facilitator) | NM_015367 | chr22 | -1.45 | down | 0.036 |
| SLC5A9 | solute carrier family 5 (sodium/sugar cotransporter), member 9 | NM_001011547 | chr1 | 1.70 | up | 0.036 |
| SAA4 | serum amyloid A4, constitutive | NM_006512 | chr11 | -1.28 | down | 0.038 |
| PARD3 | par-3 family cell polarity regulator | NM_001184792 | chr10 | -1.03 | down | 0.042 |
| LSM14B | LSM14B, SCD6 homolog B (S. cerevisiae) | NM_144703 | chr20 | -1.83 | down | 0.043 |
| LEPR | leptin receptor | NM_002303 | chr1 | -1.39 | down | 0.043 |
| ILF2 | interleukin enhancer binding factor 2 | ENST00000368681 | chr1 | -1.62 | down | 0.048 |
| PRKAG1 | protein kinase, AMP-activated, gamma 1 non-catalytic subunit | NM_001206710 | chr12 | 1.05 | up | 0.049 |
| UCN | urocortin | NM_003353 | chr2 | 1.10 | up | 0.049 |


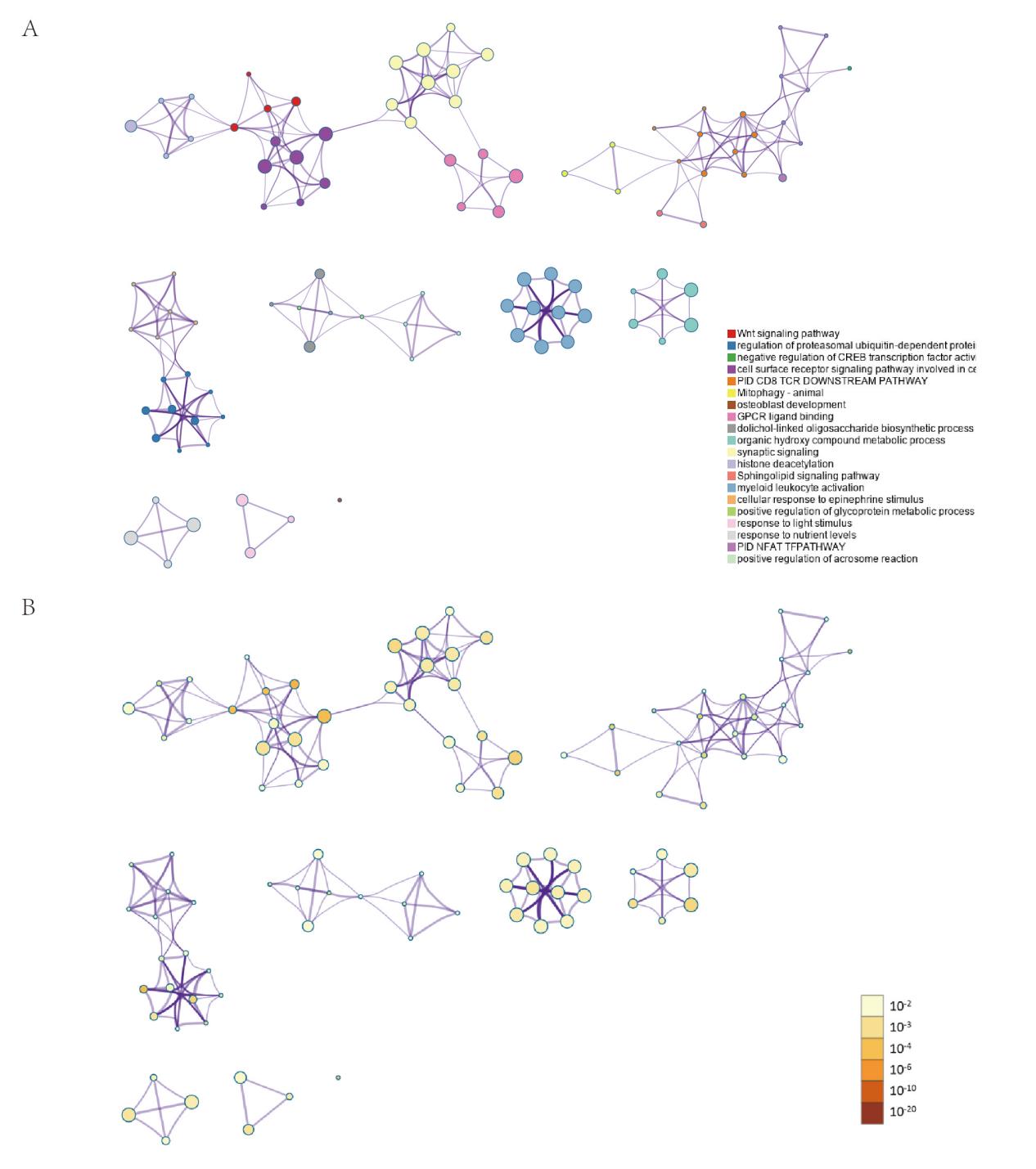


Fig.S1 Functional enrichment analysis of DEmRNAs in ICH. (A) The integration results of the top 20 most enriched pathways, Hallmark gene set, KEGG pathway, and GO biological process. Nodes were colored by cluster ID. (B) The integration results of the top 20 most enriched pathways, Hallmark gene set, KEGG pathway, and GO biological process. Nodes were colored by p-value.


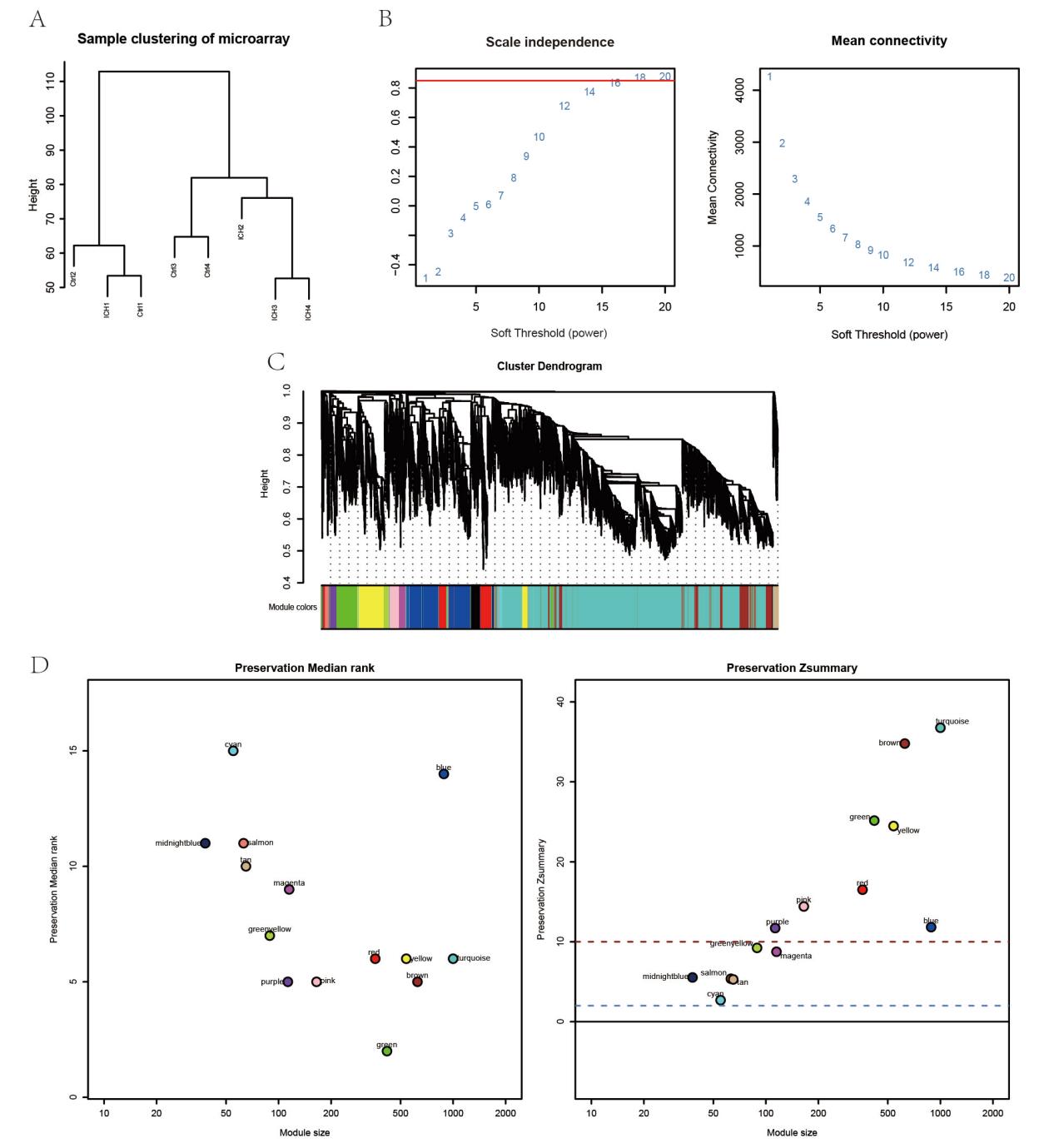


Fig.S2 Analysis of the lncRNAs modules in ICH by WGCNA. (A) The sample dendrogram displayed no outliers in our study. (B) Selection of the appropriate soft threshold. Scale independence was on the left, and mean connectivity was on the right. (C) The cluster dendrogram of co-expression network lncRNAs modules in different colors. (D) The results of preservation statistics with preservation median rank on the left and preservation Zsummary on the right. The dashed blue and red line showed the Z summary score of  2 and 10 respectively.
